# Survival disparities and predictors in gastric cancer: a population-based study from Kazakhstan (2012–2023)

**DOI:** 10.3389/fonc.2025.1670082

**Published:** 2025-11-21

**Authors:** Aidana Rakhmankulova, Yesbolat Sakko, Altay Kerimkulov, Meiram Mamlin, Sanzhar Shalekenov, Dinara Zharlyganova, Almira Manatova, Zhuldyz Kuanysh, Zhandos Burkitbayev, Abduzhappar Gaipov

**Affiliations:** 1 Department of Biomedical Sciences, School of Medicine, Nazarbayev University, Astana, Kazakhstan; 2 Department of Multidisciplinary Surgery, National Research Oncology Center, Astana, Kazakhstan; 3 Department of Science, National Research Oncology Center, Astana, Kazakhstan; 4 Department of Medicine, School of Medicine, Nazarbayev University, Astana, Kazakhstan

**Keywords:** gastric cancer, survival, epidemiology, risk factors, mortality, cancer stage, comorbidities, Kazakhstan

## Abstract

**Introduction:**

Gastric cancer remains a major global health burden, with Central Asia, Kazakhstan in particular, exhibiting high incidence and mortality. There is a lack of recent national data providing a detailed clinical picture of gastric cancer. Most reports have been limited to summary statistics on incidence or mortality, without stratification by tumor stage, histological subtype, survival, or associated comorbidities. This study addresses gaps by using national registry data to evaluate 12-year gastric cancer trends, patient characteristics, and outcomes. We aim to investigate gastric cancer epidemiology, survival, and associated risk factors in Kazakhstan for the prevention and control strategies.

**Methods:**

This retrospective cohort study analyzed 33,992 patients with gastric cancer using the Unified National Electronic Healthcare System in Kazakhstan from 2012 to 2023. Cases were identified via ICD-10 codes (C16.0-C16.9). Demographic, staging, histological, treatment and comorbidity data were extracted. Outcomes included incidence, mortality, and survival. Kaplan-Meier analysis and Cox regression were used to evaluate survival differences and predictors. Population-based rates were age-standardized using WHO standards.

**Results:**

The age-standardized incidence rate declined from 17.46 to 13.63 per 100,000; mortality dropped from 16.16 to 8.74. Prevalence doubled over 12 years. Most cases (33.5%) were diagnosed at Stage III, closely followed by Stage II and Stage IV. One- and five-year survival rates were 38.1% and 17.1%, respectively. Men and patients aged 60–69 had the highest incidence. Survival declined sharply with stage: Stage I (49.1%) vs Stage IV (3.5%, P < 0.001). The most common tumor site was the cardia, and adenocarcinoma was the predominant histology. Cox regression identified older age (HR 1.17 per decade), advanced stage (HR 3.48 for Stage IV), recurrence, metastases and cancer complications as significant mortality predictors (all P < 0.001). Cardiovascular and gastrointestinal diseases were the most common comorbidities.

**Conclusion:**

Gastric cancer in Kazakhstan shows late-stage diagnosis and poor survival. Targeted screening, earlier diagnosis, and improved management of comorbidities are essential to improve outcomes and reduce mortality.

## 1 Introduction

Gastric cancer (also called stomach cancer) is one of the most widespread cancer types worldwide. According to the GLOBOCAN (2022) data stomach cancer was 5^th^ by incidence (968,350 cases) and 5^th^ by mortality (659,853 deaths) (1). Globally, the incidence of gastric cancer increased from 883 thousand cases to 1.3 million between 1990 and 2019. In 2017, global age-standardized incidence rate (ASIR) was 15.36 while age-standardized mortality rate (ASMR) was 10.98 per 100,000 people. In 2017, Central Asia reported an ASIR of 14.12 and a nearly equivalent ASMR of 14.34 per 100,000 population, indicating not only a high disease burden but also a high case-fatality rate (2).

According to the Global Cancer Observatory, Asia reported the highest incidence, mortality, and prevalence of gastric cancer for both sexes in 2022 (3). Globally, the age-standardized incidence rate (ASIR) for gastric cancer was 9.2 per 100,000 population (12.8 in men and 6.0 in women), while the corresponding mortality rate was 6.1 (8.6 in men and 3.9 in women) (4). In Kazakhstan, the ASMR decreased from 16.4 per 100,000 in 2009 to 9.4 in 2018, reflecting progress in disease control (5). However, a 2016 predictive model anticipated a continued increase in gastric cancer burden in the country (6). Between 2014 and 2022, gastric cancer had the second-highest prevalence after respiratory cancers, representing 11.4% of all cancer types (7).

Known risk factors for gastric cancer are: lifestyle and diet, genetic predisposition, medical conditions, applied treatment, demographic characteristics, occupational exposures and radiation (8). Late-stage diagnosis and poor early detection remain key drivers of high mortality.

While previous studies have described general cancer trends in Kazakhstan (7, 9), there is a lack of recent national data providing a detailed clinical picture of gastric cancer as the latest one published covers the period from 2005 till 2014 (10) and 2009-2018 (5)Recent reports have been limited to summary statistics in local journals, newsletters on incidence or mortality, without stratification by tumor stage, histological subtype, survival, or associated comorbidities (11). No study to date has used large-scale electronic health records to analyze long-term trends alongside clinical outcomes at the population level. This study addresses that gap by providing the first comprehensive nationwide analysis of gastric cancer in Kazakhstan using individual-level data over a 12-year period (2012-2023). By combining demographic, clinical, and survival information, we aim to identify diagnostic delays, characterize high-risk patient groups, and support the development of targeted screening and treatment strategies.

## 2 Methods

### 2.1 Study design and database

This retrospective, population-based study aimed to investigate the epidemiology, comorbidities, histological subtypes, and staging of gastric cancer in Kazakhstan. Data were sourced from the UNEHS’s Electronic Registry of Inpatients; a national digital database launched in late 2013 to consolidate inpatient medical records across Kazakhstan’s healthcare institutions (13). Historical data from prior years were manually integrated into the system, with the 84 most complete and consistent coverage beginning in 2012. Also, data was taken from the Electronic Registry of Oncological Patients (EROP) in particular, between 2012 and 2023, which records clinically relevant encounters (inpatient admissions, outpatient visits, and official follow-up) across 3 participating public facilities (12). Patients in the registry are assigned unique, lifelong population registry numbers (RpnIDs), ensuring that no identifiable information is available. 

Population denominators for rate calculations were obtained from the Statistics Committee under the Ministry of National Economy of the Republic of Kazakhstan (14).

### 2.2 Participants and eligibility criteria

Gastric cancer cases were identified using ICD-10 codes C16, C16.0-C16.9. A total of 95,005 hospital admission records from 2012 to 2023 were initially extracted. After data cleaning and de-duplication based on unique registry personal numbers (RpnID), 33,992 unique patients were retained for analysis.

### 2.3 Exposures and covariates

Individual patient data included RpnID, demographic and clinical characteristics.

There were more than 100 nationalities; therefore, ethnicity was grouped as Kazakhs, Russians, Ukrainians, and Others. The year of registration was determined based on the patient’s initial admission. Age was categorized into eight groups (0-19, 20-29,…, 80+ years), adapted from the Centers for Disease Control and Prevention for epidemiological analysis (15).

Occupational status was divided into 19 groups. This structured approach ensures clarity and consistency in the dataset while allowing for comprehensive epidemiological analysis.

The timing of comorbidity diagnosis was determined using the admission date associated with each ICD-10-coded diagnosis in the UNEHS inpatient registry. “Before cancer” refers to comorbidities recorded in any inpatient admission prior to the date of the first gastric cancer admission. “Simultaneous” was defined as comorbidities recorded during the same hospitalization as the first gastric cancer diagnosis. “After cancer” refers to comorbidities first recorded in subsequent admissions following the initial cancer admission. Due to the lack of outpatient data and treatment timelines, this classification may be affected by reporting delays or hospitalization priorities.

### 2.4 Tumor characteristics

Tumor staging followed the TNM classification system. Due to inconsistencies in the national clinical protocol (now under revision), staging was cross-validated using the Russian Federation’s guidelines, which align with the American Cancer Society standards (16–18). In Kazakhstan, gastric cancer staging is standardized nationally and follows the Russian Federation clinical protocol, which is aligned with the TNM 8th edition. As a result, staging was consistently applied across institutions during the study period, and no retrospective restaging or conversion using ICD-O or other systems was required. Stage migration was not anticipated, given the uniform use of the TNM-based classification system throughout. For analysis cancer stages were grouped into bigger ones (*In Situ*, I, II, III, IV) due to lack of precise diagnosis written in database. Histological types of tumors were presented in more than 100 types and were grouped into 10 categories. Comorbidities were collected by the RPN IDs from all databases and included into the analysis. Overall, 49 comorbidities were identified.

We calculated and report the proportion of missing values for key variables including histology, stage, and comorbidity status. No imputation was performed; analyses were conducted using complete case data, with “unknown” categories retained as separate groups where applicable. Distribution of missing data is also provided in the results.

### 2.5 Outcome assessment

Incidence proportion, prevalence and mortality rates based on the admission and discharge status were calculated based on the following formulas (1–3):

Age-standardized incidence (ASIR) per 100,000 people =

(1) $$\frac{\sum (\;\frac{Cases_{i}}{Population_{i}}\;\times \;100,000\times StandardPop_{i})}{1,000,000}$$

Age-standardized mortality rate (ASMR) per 100,000 people =

(2) $$\frac{\sum (\;\frac{Death_{i}}{Population_{i}}\;\times \;100,000\times StandardPop_{i})}{1,000,000}$$

Age-standardized prevalence per 100,000 people =

(3) $$\frac{ASIR_{t-1}\;-\;ASMR_{t-1}\;+\;ASIR_{t}}{1,000,000}$$

where, i = age group, t = current year, StandardPop = WHO standard population per age group.

Standard population age-group data was taken from the WHO official report for standard population numbers from 2000 to 2025.

In the survival analysis, the start date was defined as the day of the first admission, and the follow-up was until October 11, 2024 (day of registry download), or until the date of death.

### 2.6 Statistical analysis

Data cleaning and statistical analysis were performed using Stata 18.5 MP version (19). Descriptive statistics summarized patient demographics and clinical features. Continuous variables were reported as means with standard deviations, and categorical variables as frequencies and percentages. Kaplan–Meier survival curves were used to estimate overall survival by cancer stage at diagnosis. To identify factors associated with mortality, we applied Cox proportional hazards regression. Variables for the multivariable Cox regression model were selected using a stepwise backward elimination approach, based on Akaike Information Criterion (AIC). Candidate variables included those with clinical relevance and statistical significance (P < 0.05) in univariate analysis. Multicollinearity was assessed using variance inflation factors (VIFs), and no variables exceeded accepted thresholds. Because treatment variables (surgery, chemotherapy, radiotherapy) were >60% missing and lacked timing, we did not include them as baseline covariates to avoid selection and immortal-time bias. To partially account for prognosis that influences treatment selection, the multivariable Cox model adjusted for stage, metastasis/recurrence status, comorbidity burden and timing (relative to cancer), age, sex, and histology. Although some variables - such as comorbidities and complications - may be interrelated, their inclusion was justified based on their clinical importance and distinct coding in the registry data. Both univariable and multivariable models were constructed, with results reported as hazard ratios (HRs) and adjusted hazard ratios (AHRs) with 95% confidence intervals. Statistical significance was set at ɑ < 0.05.

## 3 Results

**Figure 1:**
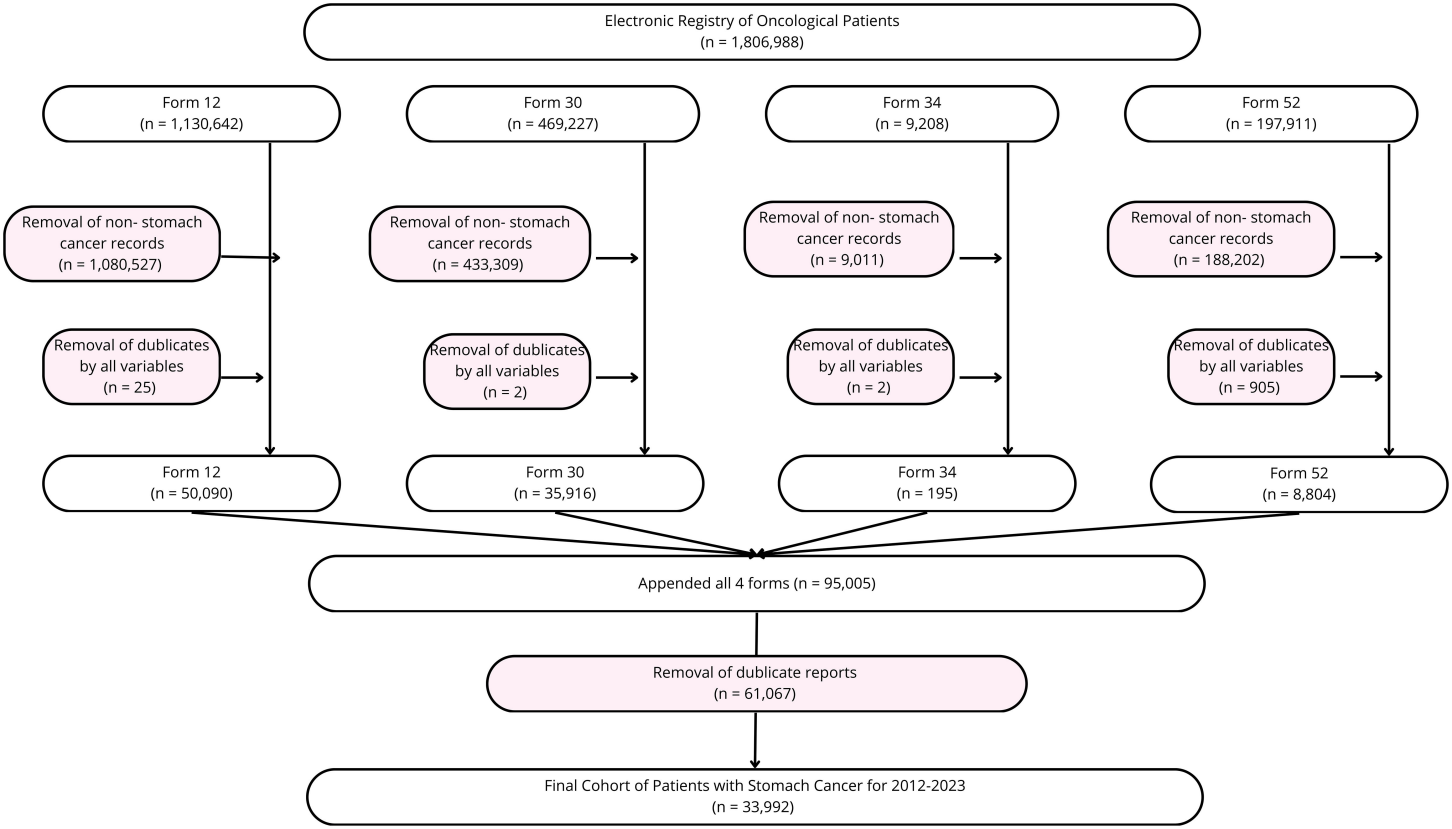
Data cleaning process flow chart.

**Figure 1** shows the data cleaning and data collection process. Overall, 1,806,988 patient records were obtained and 33,992 used for further analysis.

### 3.1 Demographic data

The demographic data is summarized in **Table 1**. In total 33,992 (35.9% women and 64.1% men) gastric cancer patient records were identified and analyzed in the national electronic database for the period 2012-2023, from all Kazakhstani regions. The mean age (± SD) was 64.2 ± 11.5 years. Of the patients, 59.3% were Kazakhs, 24.5% were Russians, 3.7% were Ukrainians, and 12.6% were listed as other nationalities.

**Table 1:** Demographic and clinical characteristics of the cohort.

Factor	Total	Alive	Deceased
Total	33992	5881 (17.3%)	28111 (82.7%)
Age Group			
0-19	16 (<1%)	4 (0.1%)	12 (<1%)
20-29	203 (0.6%)	37 (0.6%)	166 (0.6%)
30-39	859 (2.5%)	214 (3.6%)	645 (2.3%)
40-49	2598 (7.6%)	582 (9.9%)	2016 (7.2%)
50-59	7518 (22.1%)	1608 (27.3%)	5910 (21.0%)
60-69	11717 (34.5%)	2203 (37.5%)	9514 (33.8%)
70-79	8739 (25.7%)	1048 (17.8%)	7691 (27.4%)
above 80	2342 (6.9%)	185 (3.1%)	2157 (7.7%)
Gender			
female	12203 (35.9%)	2411 (41.0%)	9792 (34.8%)
male	21789 (64.1%)	3470 (59.0%)	18319 (65.2%)
Year			
2012	2680 (7.9%)	203 (3.5%)	2477 (8.8%)
2013	2771 (8.2%)	253 (4.3%)	2518 (9.0%)
2014	2731 (8.0%)	277 (4.7%)	2454 (8.7%)
2015	2901 (8.5%)	307 (5.2%)	2594 (9.2%)
2016	2917 (8.6%)	483 (8.2%)	2434 (8.7%)
2017	2872 (8.4%)	430 (7.3%)	2442 (8.7%)
2018	2899 (8.5%)	467 (7.9%)	2432 (8.7%)
2019	2935 (8.6%)	599 (10.2%)	2336 (8.3%)
2020	2656 (7.8%)	475 (8.1%)	2181 (7.8%)
2021	2787 (8.2%)	601 (10.2%)	2186 (7.8%)
2022	2991 (8.8%)	755 (12.8%)	2236 (8.0%)
2023	2850 (8.4%)	1031 (17.5%)	1819 (6.5%)
Stage at the diagnosis			
I	3765 (11.1%)	1808 (30.7%)	1957 (7.0%)
II	9909 (29.2%)	2245 (38.2%)	7664 (27.3%)
III	11378 (33.5%)	1358 (23.1%)	10020 (35.6%)
IV	8259 (24.3%)	323 (5.5%)	7936 (28.2%)
In Situ	592 (1.7%)	142 (2.4%)	450 (1.6%)
missing	89 (0.3%)	5 (0.1%)	84 (0.3%)
ICD-10 code			
C16 (Non-differentiated Stomach Cancer)	4604 (13.5%)	332 (5.6%)	4272 (15.2%)
C16.0 (Malignant neoplasm (MN) of cardia)	10468 (30.8%)	1933 (32.9%)	8535 (30.4%)
C16.1 (MN of fundus of stomach)	829 (2.4%)	143 (2.4%)	686 (2.4%)
C16.2 (MN of body of stomach)	8482 (25.0%)	1658 (28.2%)	6824 (24.3%)
C16.3 (MN of antrum)	1893 (5.6%)	485 (8.2%)	1408 (5.0%)
C16.4 (MN of pylorus)	1406 (4.1%)	343 (5.8%)	1063 (3.8%)
C16.5 (MN of lesser curvature, unspecified)	197 (0.6%)	45 (0.8%)	152 (0.5%)
C16.6 (MN of greater curvature, unspecified)	148 (0.4%)	36 (0.6%)	112 (0.4%)
C16.8 (MN of overlapping lesions of stomach)	2061 (6.1%)	130 (2.2%)	1931 (6.9%)
C16.9 (MN of stomach, unspecified)	1084 (3.2%)	138 (2.3%)	946 (3.4%)
mixed	2820 (8.3%)	638 (10.8%)	2182 (7.8%)
Histological type of tumor			
adenocarcinoma	20944 (61.7%)	3513 (59.8%)	17431 (62.1%)
other carcinomas	3687 (10.8%)	442 (7.5%)	3245 (11.5%)
other	6337 (18.6%)	1261 (13.9%)	5076 (19.6%)
sarcomas	71 (0.2%)	42 (0.7%)	29 (0.1%)
squamous cell carcinoma	1053 (3.1%)	123 (2.1%)	930 (3.3%)
missing	1900 (5.6%)	942 (16.0%)	958 (3.4%)
Metastases. Recurrence			
metastases	8877 (26.1%)	610 (10.4%)	8267 (29.4%)
metastases and recurrence	196 (0.6%)	11 (0.2%)	185 (0.7%)
no metastases and no recurrence	10782 (31.7%)	3325 (56.5%)	7457 (26.5%)
no recurrence	13740 (40.4%)	1881 (32.0%)	11859 (42.2%)
recurrence	397 (1.2%)	54 (0.9%)	343 (1.2%)
Surgery type			
No operation/Missing	26074 (76.9%)	3732 (63.5%)	22315 (79.6%)
Extended Gastrectomy	2165 (6.4%)	617 (10.5%)	1548 (5.5%)
Gastrectomy (total/subtotal/partial/local)	3213 (9.5%)	1216 (20.7%)	1997 (7.1%)
Esophageal Surgery	285 (0.8%)	123 (2.1%)	162 (0.6%)
HPB/Intestine/Spleen	389 (1.1%)	39 (0.7%)	350 (1.2%)
Reconstructions/access/support	1802 (5.3%)	149 (2.5%)	1653 (5.9%)
Treatment			
Systemic	8124 (23.9%)	2218 (37.7%)	5906 (21.1%)
Surgery	33 (0.1%)	2 (<1%)	31 (0.1%)
Chem-radio therapy	151 (0.4%)	23 (0.4%)	128 (0.5%)
Radio therapy	3297 (9.7%)	693 (11.8%)	2604 (9.3%)
Missing	22323 (65.8%)	2940 (50.0%)	19356 (69.1%)
Count of comorbidity categories present			
0 or missing	12124 (35.5%)	841 (13.8%)	11283 (40.1%)
1	10641 (31.4%)	2019 (34.4%)	8605 (30.7%)
2	5974 (17.6%)	1402 (23.9%)	4564 (16.3%)
3	2785 (8.2%)	777 (13.2%)	2007 (7.2%)
4	1417 (4.2%)	478 (8.1%)	938 (3.3%)
5	604 (1.8%)	207 (3.5%)	397 (1.4%)
6	283 (0.8%)	116 (2.0%)	167 (0.6%)
7	93 (0.3%)	44 (0.7%)	49 (0.2%)
8	35 (0.1%)	15 (0.3%)	20 (0.1%)
9	15 (<1%)	5 (0.1%)	10 (<1%)
10	11 (<1%)	3 (0.1%)	8 (<1%)
11	6 (<1%)	0 (0.0%)	6 (<1%)
12	4 (<1%)	1 (<1%)	3 (<1%)
Comorbidities			
Gastrointestinal Diseases (total)	7641 (22.48%)	1870 (31.8%)	5771 (20.5%)
Gastrointestinal diseases	6607 (19.44%)	1548 (26.32%)	5059 (18.0%)
Cholecystitis	1680 (4.94%)	541 (9.2%)	1139 (4.05%)
Cardiovascular Diseases (total)	7142 (21.01%)	1729 (29.4%)	5413 (19.3%)
Hypertension	4489 (13.21%)	905 (15.39%)	3584 (12.75%)
Angina pectoris	1627 (4.79%)	535 (9.1%)	1092 (3.88%)
Cerebrovascular disease	1291 (3.8%)	399 (6.78%)	892 (3.17%)
Coronary artery disease	1220 (3.59%)	240 (4.08%)	980 (3.49%)
Congestive heart failure	972 (2.86%)	282 (4.8%)	690 (2.45%)
Peripheral artery disease	266 (0.78%)	90 (1.53%)	176 (0.63%)
Thromboembolism	245 (0.72%)	82 (1.39%)	163 (0.58%)
Myocardial infarction	175 (0.51%)	61 (1.04%)	114 (0.41%)
Atrial fibrillation	143 (0.42%)	37 (0.63%)	106 (0.38%)
Arrhythmia	124 (0.36%)	38 (0.65%)	86 (0.31%)
Valvular heart disease	43 (0.13%)	12 (0.2%)	31 (0.11%)
Hematologic & Renal Diseases (total)	4151 (12.21%)	1010 (17.2%)	3141 (11.2%)
Anemia	2176 (6.4%)	522 (8.88%)	1654 (5.88%)
Renal	2101 (6.18%)	536 (9.11%)	1565 (5.57%)
Chronic kidney disease	279 (0.82%)	61 (1.04%)	218 (0.78%)
Electrolyte disorder	20 (0.06%)	4 (0.07%)	16 (0.06%)
Infectious Diseases & Immune Disorders (total)	4092 (12.04%)	1376 (23.4%)	2716 (9.7%)
Infectious diseases	1514 (4.45%)	577 (9.81%)	937 (3.33%)
Other cancer types	1312 (3.86%)	476 (8.09%)	836 (2.97%)
Neoplasms	676 (1.99%)	285 (4.85%)	391 (1.39%)
Cancer complications	172 (0.51%)	14 (0.24%)	158 (0.56%)
HIV	23 (0.07%)	9 (0.15%)	14 (0.05%)
Dermatological & Sensory Disorders (total)	2750 (8.09%)	839 (14.3%)	1911 (6.8%)
Ophthalmological	2037 (5.99%)	638 (10.85%)	1399 (4.98%)
Dermatological	648 (1.91%)	185 (3.15%)	463 (1.65%)
Otolaryngological	184 (0.54%)	51 (0.87%)	133 (0.47%)
Respiratory Diseases (total)	2451 (7.21%)	680 (11.6%)	1771 (6.3%)
Pneumonia	851 (2.5%)	290 (4.93%)	561 (2.0%)
Tuberculosis	766 (2.25%)	133 (2.26%)	633 (2.25%)
Other respiratory	583 (1.72%)	214 (3.64%)	369 (1.31%)
Chronic obstructive pulmonary disease	455 (1.34%)	107 (1.82%)	348 (1.24%)
Pulmonary embolism	40 (0.12%)	6 (0.1%)	34 (0.12%)
Genitourinary & Reproductive Disorders (total)	2340 (6.88%)	682 (11.6%)	1658 (5.9%)
Urological	1510 (4.44%)	426 (7.24%)	1084 (3.86%)
Gynecological	539 (1.59%)	185 (3.15%)	354 (1.26%)
Pregnancy related	410 (1.21%)	107 (1.82%)	303 (1.08%)
Musculoskeletal & Connective Tissue Disorders (total)	2251 (6.62%)	768 (13.1%)	1483 (5.3%)
Connective tissue disorders/musculoskeletal disorders	1879 (5.53%)	647 (11.0%)	1232 (4.38%)
Osteoarthritis	571 (1.68%)	183 (3.11%)	388 (1.38%)
Neurological & Mental Health Disorders (total)	2344 (6.90%)	673 (11.4%)	1671 (5.9%)
Neurological	1942 (5.71%)	610 (10.37%)	1332 (4.74%)
Mental health	321 (0.94%)	52 (0.88%)	269 (0.96%)
Assault, self-harm	41 (0.12%)	10 (0.17%)	31 (0.11%)
Metabolic & Endocrine Disorders (total)	1951 (5.74%)	361 (6.1%)	1590 (5.7%)
Diabetes	1608 (4.73%)	277 (4.71%)	1331 (4.73%)
Thyroid disorder	206 (0.61%)	66 (1.12%)	140 (0.5%)
Obesity	130 (0.38%)	30 (0.51%)	100 (0.36%)
Malnutrition	119 (0.35%)	21 (0.36%)	98 (0.35%)
Weight loss	2 (0.01%)	1 (0.02%)	1 (0.0%)
Liver & Pancreatic Diseases (total)	1884 (5.54%)	523 (8.9%)	1361 (4.8%)
Liver & Pancreatic diseases	1775 (5.22%)	493 (8.38%)	1282 (4.56%)
Hepatitis	123 (0.36%)	36 (0.61%)	87 (0.31%)
Toxicology & Miscellaneous Conditions (total)	3623 (10.66%)	808 (13.7%)	2815 (10.0%)
Injuries & trauma	443 (1.3%)	182 (3.09%)	261 (0.93%)
Alcohol disorder	437 (1.29%)	75 (1.28%)	362 (1.29%)
Drug use	425 (1.25%)	66 (1.12%)	359 (1.28%)
Poisoning	28 (0.08%)	11 (0.19%)	17 (0.06%)

**Figure 2:**
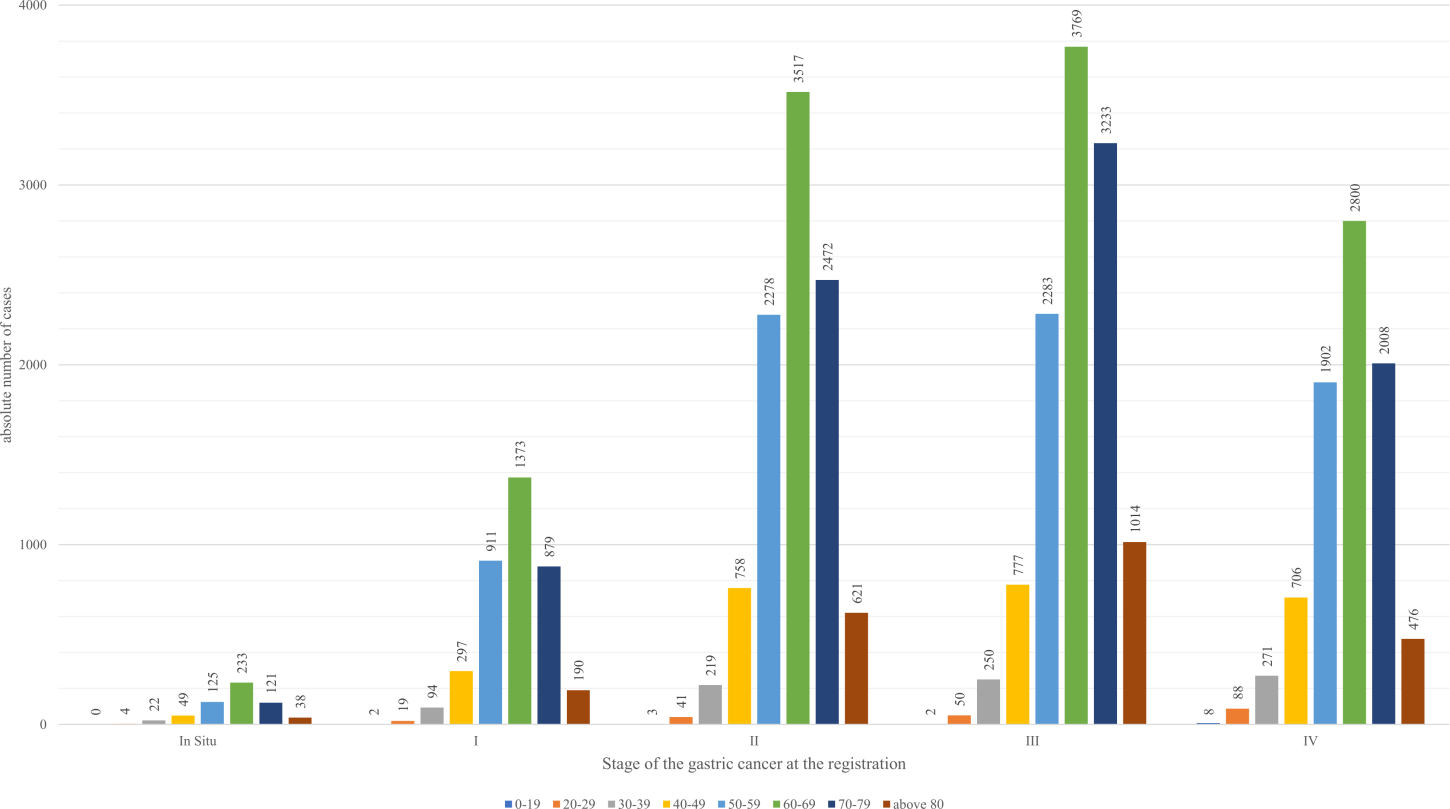
Prevalence of gastric cancer cases by age groups and stage (2012-2023).

Among the age groups, as seen from **Figure 2** 60-69-year-olds had the highest registered cases of gastric cancer within the period, and most of the registrations occurred at Stage III of the disease (33.47%).

**Figure 3:**
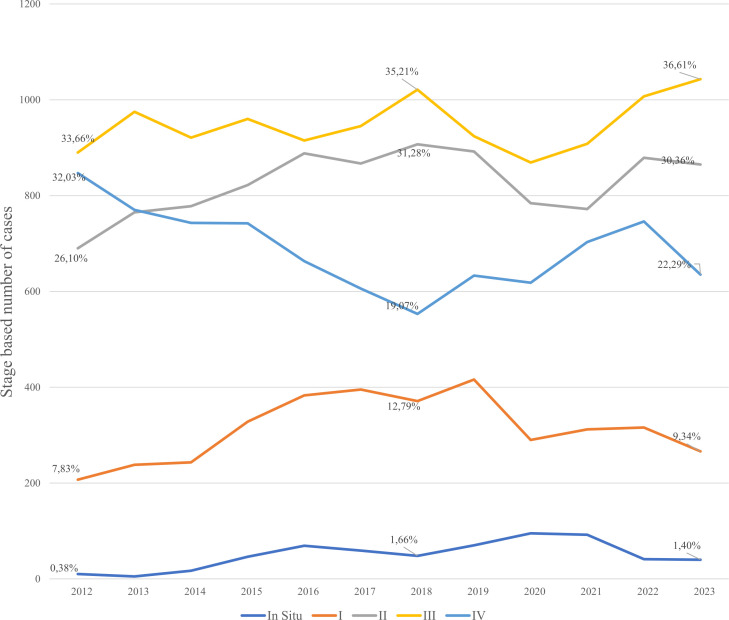
Absolute number of new cases occurrence by the year and stage.

The annual number of registered gastric cancer cases remained relatively stable, ranging from 2,644 cases in 2012 to a peak of 2,989 in 2022, with a temporary decline in 2020 (2,656 cases) likely due to COVID-19–related disruptions in healthcare services (**Figure 3**). Stage III consistently accounted for the highest proportion of diagnoses, increasing from 33.6% in 2012 to 36.6% in 2023, highlighting the persistent burden of late-stage detection. Stage IV cases declined from 32.0% to 22.3%, while Stage II diagnoses increased from 26.1% to 30.4% over the same period. The proportion of early-stage (Stage I) cases remained lowest throughout, underscoring the ongoing challenge of early detection.

**Figure 4:**
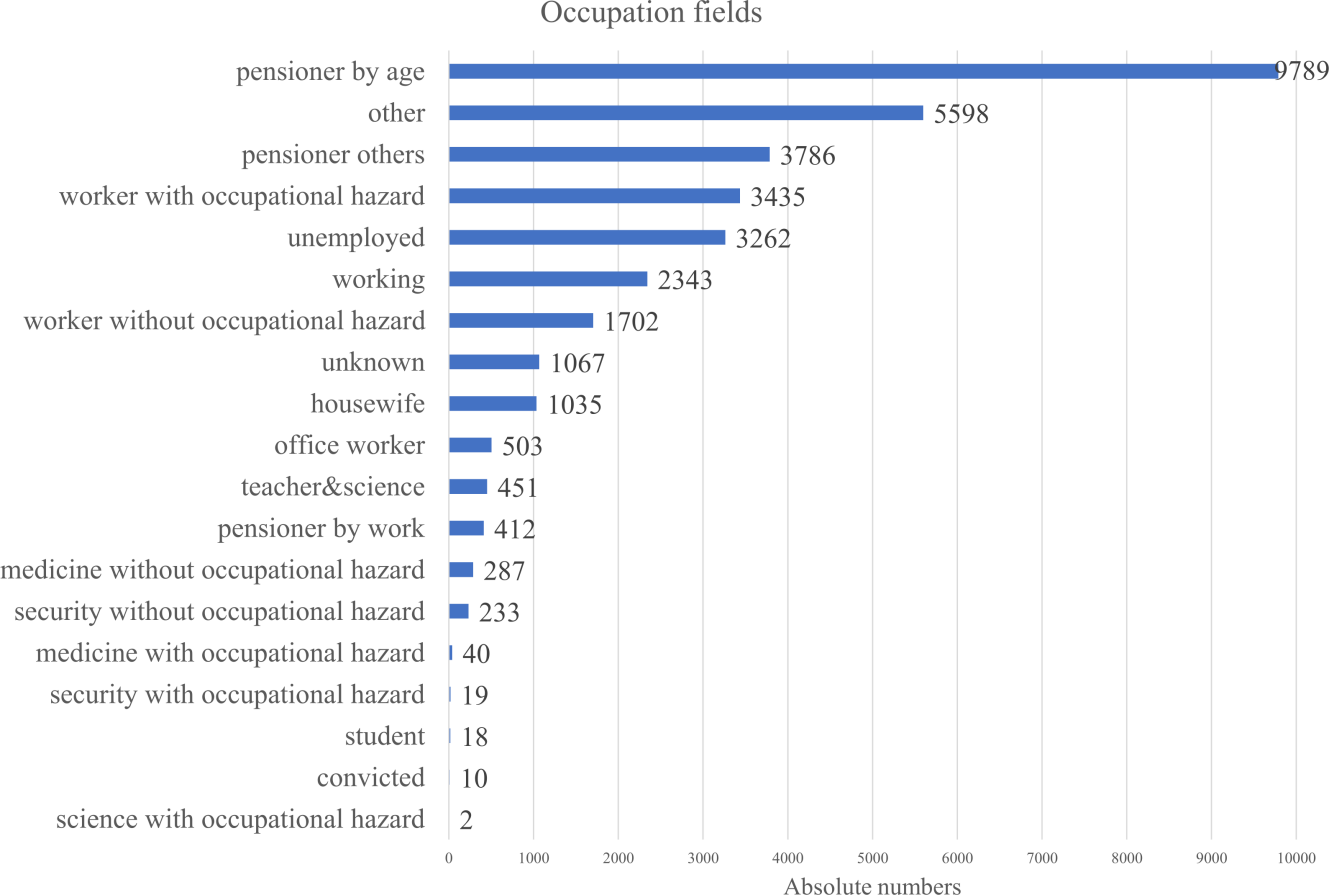
Occupational distribution of patients with gastric cancer.

Describing the social status of the cohort, information wasn’t very consistent throughout the database, that is why we merged any information on the person, regarding their occupation into the **Figure 4**. The analysis of occupational backgrounds among gastric cancer patients revealed that the largest proportion were individuals retired by age, accounting for the majority of cases. This was followed by those categorized under “other” occupations and pensioners from unspecified fields. A notable share of patients were workers exposed to occupational hazards, suggesting potential environmental or workplace-related risk factors. The term worker with occupational hazard included workers from fields of transport, water transport, clerical, woodworking industry, railway worker, industry, construction, leather worker, public utilities and household services worker, forestry, machinery, metallurgy, miner, petrochemical industry, shoemaker, food industry worker, manufacturing, extramural worker, agricultural industry, glass and porcelain industry, building materials, chemical industry, sewing worker. These findings highlight a strong representation of older and retired populations, as well as possible links between occupational exposures and gastric cancer incidence.

### 3.2 Clinical data

The most common histological type was adenocarcinoma (61.6%), accounting for 62.0% of all deaths. Rare subtypes such as neuroendocrine carcinoma (0.5%) and sarcomas (0.2%) showed varied survival rates due to low case counts.

Metastases were present in 26.1% of cases and associated with a high mortality rate (29.4%, 8,267 cases). Patients with both metastases and recurrence (0.6%) had the highest mortality (94.4%). In contrast, those with no metastases or recurrence had improved outcomes, with 32.5% surviving, while patients with recurrence alone (1.2%) still faced high mortality (86.4%) (**Table 1**).

### 3.3 Treatment

Treatment of stomach cancer is based on the Kazakhstan’s protocol of treatment published in 2022 year (17), and also in accordance with NCCN 2025 (20) and Japanese guideline 2022 (21). Based on the Kazakhstani protocol, the sections on stage-based treatment state: for stages 0-II - subtotal/total gastrectomy (EMR for Tis/T1a) with mandatory D2 lymph node dissection; for locally advanced T3-T4 or N1-N2 - curative surgery plus 2–3 cycles of neoadjuvant chemotherapy and adjuvant polychemotherapy; for stage IV - palliative operations only (no lymphadenectomy) and palliative chemotherapy; and for recurrence - individualized surgery/endoscopic options plus palliative chemotherapy (17).

Based on the results obtained from the database (**Table 1**) most patients had no recorded surgery or data was missing (26,136, or 76.9%). It wasn’t possible to differentiate them, so they are written as one category “no operation/missing”. Among specific procedures, 3 213 (9.5%) underwent gastrectomy (total/subtotal/partial/local), 2 165 (6.4%) extended gastrectomy, 1 802 (5.3%) reconstructive/access procedures, 389 (1.1%) HPB/intestine/spleen procedures, and 285 (0.8%) esophageal surgery. **Table 2** shows the distribution of type of surgery done by stages of disease in patients. It was seen that the likelihood of having no operation increased with advancing stage: 73.1% in *In Situ* and 66.6%–67.9% in stages I–II, rising to 78.3% in stage III and 90.5% in stage IV. Gastrectomy was comparatively concentrated in earlier stages (20.3% of stage I and 15.0% of stage II) and became uncommon in stage IV (1.3%). Reconstructive/access procedures were recorded in 3.4%–4.6% of stages I–II and ~6.1% of stages III–IV. Esophageal and HPB/intestine/spleen operations were rare across all stages (each ~1% per stage). With respect to non-surgical therapy, 8–186 patients (≈24%) had systemic therapy coded, 3 297 (9.7%) radiotherapy, 151 (0.4%) chemoradiotherapy, and 33 (0.1%) surgery-only treatment. Treatment coding was missing in 22 323 (65.8%) overall, and missingness increased with stage (57.9% in *In Situ*; 54.6% stage I; 55.1% stage II; 68.1% stage III; 81.1% stage IV).

**Table 2:** Surgery and treatment data by stage.

Factor	Total	In Situ	I	II	III	IV
N	33992	592	3765	9909	11378	8259
Surgery type						
No operation/Missing	26136 (76.9%)	433 (73.1%)	2507 (66.6%)	6726 (67.9%)	8908 (78.3%)	7475 (90.5%)
Extended Gastrectomy	2165 (6.4%)	63 (10.6%)	286 (7.6%)	990 (10.0%)	742 (6.5%)	84 (1.0%)
Gastrectomy (total/subtotal/partial/local)	3213 (9.5%)	62 (10.5%)	766 (20.3%)	1482 (15.0%)	796 (7.0%)	107 (1.3%)
Esophageal Surgery	285 (0.8%)	5 (0.8%)	48 (1.3%)	141 (1.4%)	85 (0.7%)	6 (0.1%)
HPB/Intestine/Spleen	389 (1.1%)	9 (1.5%)	31 (0.8%)	114 (1.2%)	149 (1.3%)	86 (1.0%)
Reconstructions/access/support	1802 (5.3%)	20 (3.4%)	127 (3.4%)	456 (4.6%)	698 (6.1%)	501 (6.1%)
Treatment						
Systemic	8186 (23.9%)	165 (27.9%)	1315 (34.9%)	3257 (32.9%)	2553 (22.4%)	836 (10.1%)
Surgery	33 (0.1%)	1 (0.2%)	1 (<1%)	18 (0.2%)	9 (0.1%)	4 (<1%)
Chem-radio therapy	151 (0.4%)	1 (0.2%)	26 (0.7%)	57 (0.6%)	48 (0.4%)	19 (0.2%)
Radio therapy	3297 (9.7%)	82 (13.9%)	369 (9.8%)	1119 (11.3%)	1023 (9.0%)	704 (8.5%)
Missing	22323 (65.8%)	343 (57.9%)	2054 (54.6%)	5458 (55.1%)	7745 (68.1%)	6696 (81.1%)
Count of comorbidity categories present						
0 or missing	12122 (35.5%)	135 (22.8%)	799 (21.2%)	3149 (31.8%)	4257 (37.4%)	3722 (45.1%)
1	10641 (31.4%)	207 (35.0%)	1176 (31.2%)	3294 (33.2%)	3528 (31.0%)	2419 (29.3%)
2	5974 (17.6%)	126 (21.3%)	855 (22.7%)	1813 (18.3%)	1943 (17.1%)	1229 (14.9%)
3	2785 (8.2%)	64 (10.8%)	442 (11.7%)	856 (8.6%)	910 (8.0%)	512 (6.2%)
4	1417 (4.2%)	29 (4.9%)	268 (7.1%)	457 (4.6%)	435 (3.8%)	227 (2.7%)
5	604 (1.8%)	20 (3.4%)	122 (3.2%)	190 (1.9%)	183 (1.6%)	89 (1.1%)
6	283 (0.8%)	11 (1.9%)	58 (1.5%)	102 (1.0%)	73 (0.6%)	39 (0.5%)
7	93 (0.3%)	0 (0.0%)	26 (0.7%)	30 (0.3%)	27 (0.2%)	10 (0.1%)
8	35 (0.1%)	0 (0.0%)	15 (0.4%)	7 (0.1%)	8 (0.1%)	5 (0.1%)
9	15 (<1%)	0 (0.0%)	1 (<1%)	5 (0.1%)	6 (0.1%)	3 (<1%)
10	11 (<1%)	0 (0.0%)	3 (0.1%)	2 (<1%)	4 (<1%)	2 (<1%)
11	6 (<1%)	0 (0.0%)	0 (0.0%)	3 (<1%)	1 (<1%)	2 (<1%)
12	4 (<1%)	0 (0.0%)	0 (0.0%)	1 (<1%)	3 (<1%)	0 (0.0%)

### 3.4 Comorbidities

Gastrointestinal diseases were the most common comorbidities, accounting for 7,641 cases (22.48%). Cardiovascular diseases followed with 7,142 (21.01%) cases, with hypertension (13.21%), Angina pectoris (4.79%) and cerebrovascular (3.8%) diseases as the most frequent one. Hematologic and renal diseases ranked third with 4,151 cases (12.21%), driven by anemia (2,176 cases, 6.4%). Other notable categories included infectious diseases and immune (12.04%), dermatological and sensory (8.09%) and respiratory (7.21%) disorders (**Table 1**).

The number of comorbidity categories present was 0 or missing in 12–124 patients (35.5%); 1 category in 10 641 (31.4%); 2 in 5 974 (17.6%); 3 in 2 785 (8.2%); 4 in 1 417 (4.2%); and ≥5 in 1 044 (3.1%) with progressively smaller strata up to 12 (**Table 1**). Survivors had fewer “0/missing” entries and more recorded multimorbidity than those who died: 13.8% vs 40.1% had 0/missing; 51.6% of survivors vs 29.1% of decedents had ≥2 comorbidity categories, and 27.9% vs 12.8% had ≥3 categories. Comorbidities were right-skewed overall as seen from **Table 2**. “Zero or missing” made up 12–122 patients (35.5%) and became more common with advancing stage (from 22.8% in *in-situ* to 45.1% in stage IV). One category was recorded in 10 641 (31.4%) and stayed fairly stable across stages (~29–35%). Two categories were seen in 5 974 (17.6%), falling from 22.7% in stage I to 14.9% in stage IV. Higher counts were progressively less frequent: 3 categories in 2 785 (8.2%), 4 in 1 417 (4.2%), 5 in 604 (1.8%), and ≥6 in ≤0.8% each. Taken together, later stages showed fewer documented comorbidity categories and more 0/missing entries - pointing less to truly lower multimorbidity and more to under-ascertainment or under-recording in sicker patients.

### 3.5 Tumor location

The most common site was the cardia (C16.0, 30.8%), followed by the body (C16.2, ~25%). Cases involving multiple locations were grouped as mixed. Overlaps were frequent - most notably between cardia and body (28.4%), and between body and overlapping lesions (C16.2 + C16.8) in 219 cases (5.74%), indicating extensive tumor spread (**Table 3**).

**Table 3:** Overlap of gastric cancer lesions across stomach subsite ICD-10 classifications.

ICD-10	C16.1	C16.2	C16.3	C16.4	C16.5	C16.6	C16.8	C16.9
C16.0	270 (7.07%)	1085 (28.43%)	216 (5.66%)	170 (4.45%)	22 (0.58%)	58 (1.52%)	310 (8.12%)	258 (6.76%)
C16.1		164 (4.3%)	33 (0.86%)	33 (0.86%)	6 (0.16%)	14 (0.37%)	37 (0.97%)	66 (1.73%)
C16.2			158 (4.14%)	97 (2.54%)	23 (0.6%)	38 (1.00%)	219 (5.74%)	195 (5.11%)
C16.3				83 (2.17%)	10 (0.26%)	7 (0.18%)	35 (0.92%)	37 (0.97%)
C16.4					5 (0.13%)	10 (0.26%)	30 (0.79%)	33 (0.86%)
C16.5						6 (0.16%)	3 (0.08%)	3 (0.08%)
C16.6							7 (0.18%)	22 (0.58%)
C16.8								54 (1.41%)

### 3.6 Incidence, prevalence and mortality rates

**Figure 5:**
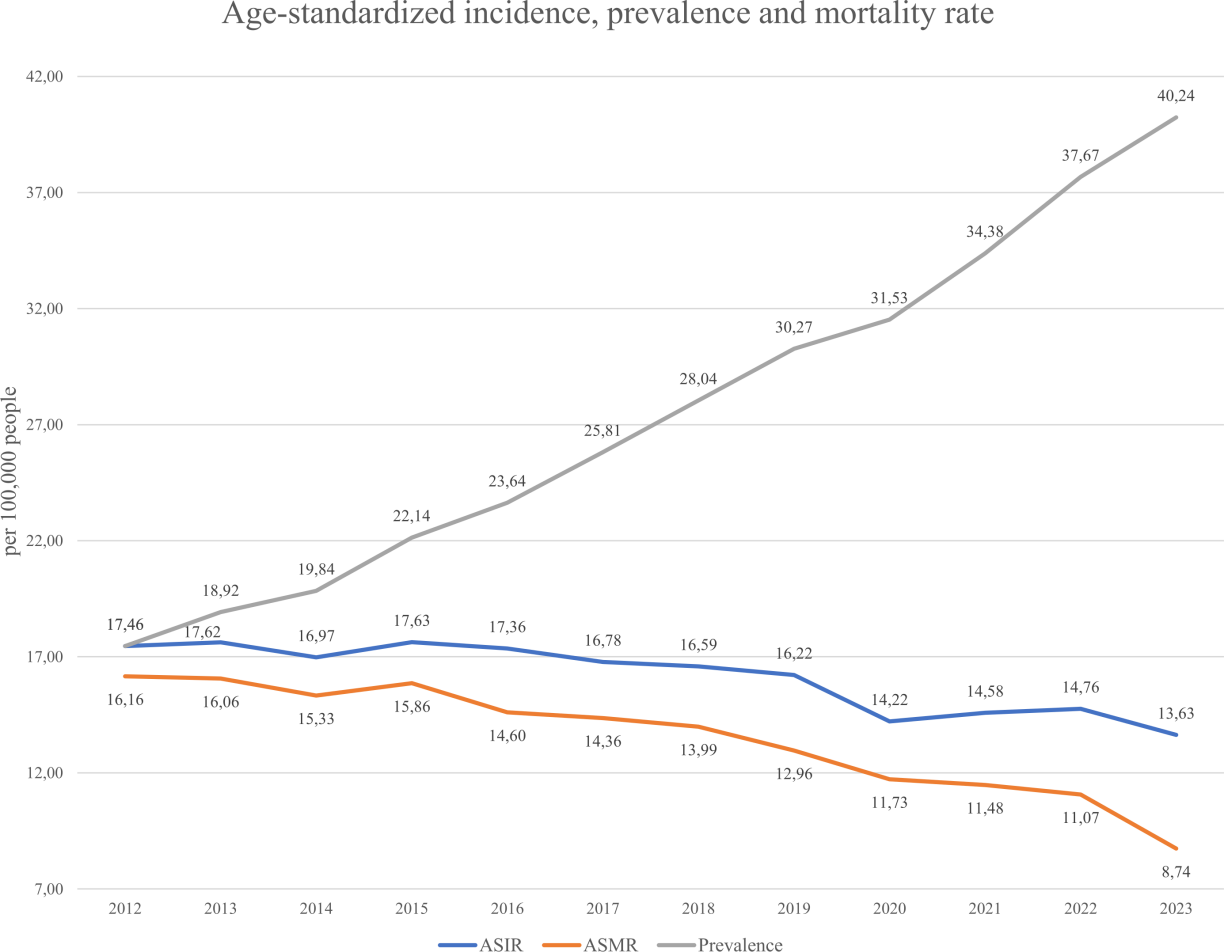
Age-standardized incidence (ASIR), prevalence and mortality (ASMR) rate from 2012-2023.

**Figure 6:**
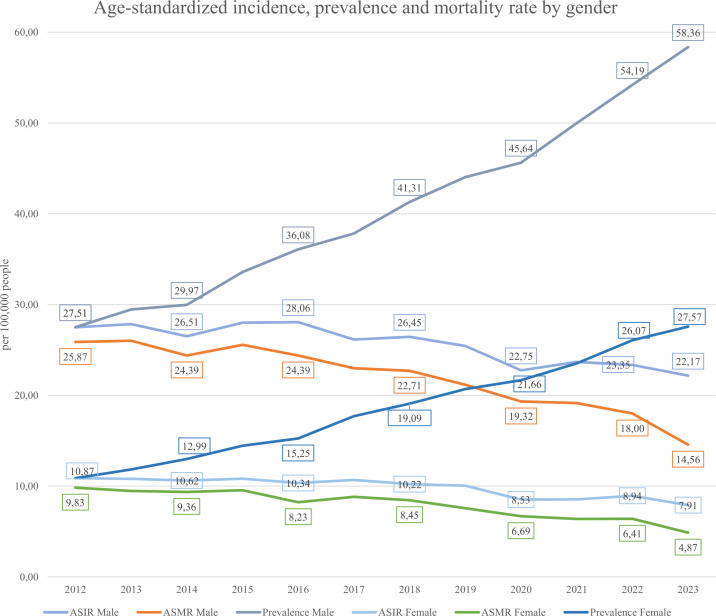
Gender-specific age-standardized incidence (ASIR), prevalence and mortality (ASMR) rate from 2012-2023.

Incidence, prevalence, and mortality are key indicators of disease trends (see Equations 1–3 in Methods). As gastric cancer is considered lifelong, all diagnosed individuals were counted as prevalent cases while alive. **Figure 5** shows that the ASIR (Age-standardized incidence Equation 1) remained relatively stable from 2012–2019 (16.06–17.46 per 100,000), then declined to 13.63 in 2023. The ASMR (Age-standardized mortality rate Equation 2) steadily decreased from 16.16 to 8.74, suggesting improvements in management or early detection. In contrast, prevalence (Equation 3) rose consistently from 17.62 to 40.24, indicating an increasing disease burden. **Figure 6** displays gender-specific trends. In males, ASIR dropped from 27.51 to 22.17, while prevalence rose sharply (10.87 to 54.19); ASMR declined from 25.87 to 14.56. In females, ASIR declined from 10.87 to 7.91, ASMR from 9.83 to 4.87, and prevalence rose from 10.87 to 27.57. Across all indicators, males bore a higher burden, with a notably sharp rise in prevalence, despite declining incidence and mortality in both sexes.

### 3.7 Survival analysis

**Figure 7:**
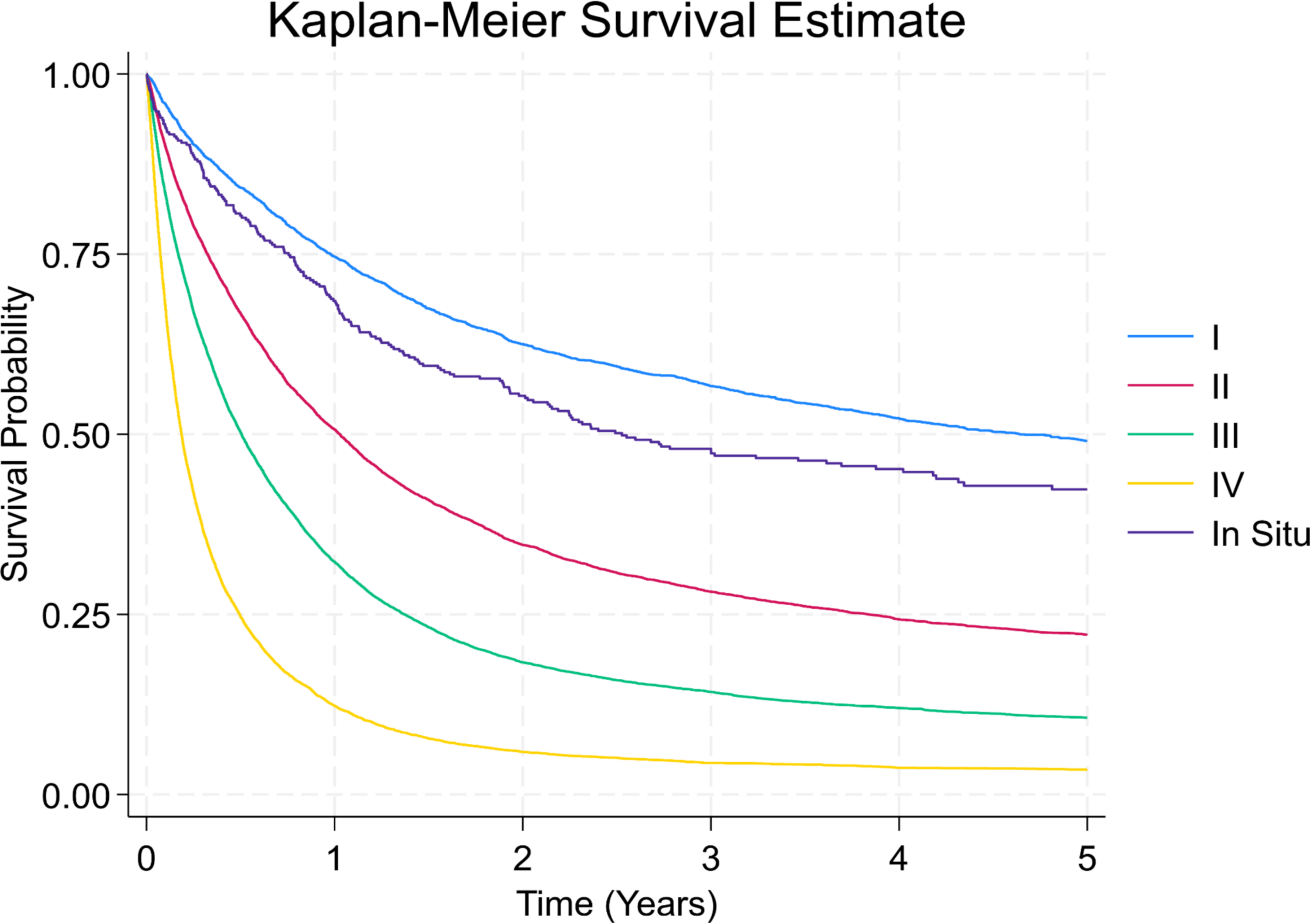
Kaplan-Meier survival curves by stage at diagnosis. This survival plot illustrates overall survival probabilities for patients with gastric cancer based on stage at diagnosis.

The Kaplan–Meier curves showed in **Figure 7** separate early and remain well ordered by stage, with the steepest early decline in stage IV and progressively flatter trajectories for stages III→I. There is no material crossing of curves, underscoring a strong stage gradient. Stage I and *in-situ* show a visible plateau after ~2–3 years, whereas stages III–IV continue to fall toward very low long-term survival. Five-year survival analysis showed 1-year survival rates declining from 74.6% (Stage I) to 12.3% (Stage IV), and 5-year survival from 49.1% to 3.5%, respectively (**Table 4**).

**Table 4:** Five-year survival rates by gastric cancer stage at diagnosis.

Time (Years)	Total	In Situ	Stage I	Stage II	Stage III	Stage IV
1	38.11%	68.5%	74.63%	50.67%	32.26%	12.31%
2	25.63%	55.34%	62.52%	34.65%	18.35%	5.91%
3	21.23%	47.98%	56.69%	28.17%	14.25%	4.39%
4	18.62%	45.17%	52.16%	24.31%	11.99%	3.7%
5	17.09%	42.34%	49.08%	22.17%	10.67%	3.45%

### 3.8 Hazard ratio by predictors

The multivariable Cox model (**Table 5**) included 31,183 patients and assessed associations between demographic and clinical variables and overall survival. In our dataset, treatment fields (surgery, chemotherapy, radiotherapy) are missing for >60% of patients, with missingness increasing with stage. Because of this extent of missing data, we did not include treatment variables in the multivariable Cox model. Treatment allocation is not random and is correlated with prognosis (confounding by indication): fitter patients are more likely to undergo curative surgery or chemotherapy, leading to downward bias in hazard estimates if treatment is unmeasured. Social status (occupational field) didn’t show any statistically significant results and thus were excluded from the model.

**Table 5:** Association between clinical variables and hazard ratio for mortality in gastric cancer patients.

Variable	Crude hazard ratio	p-value	Adjusted hazard ratio (AHR)	p-value
Age (^+10^)	1.152 (1.140-1.165)	<0.001	1.172 (1.158 – 1.185)	<0.001
Stage Category (*In Situ* ref)				
Stage I	0.814 (0.705-0.940)	0.005	0.684 (0.535-0.875)	0.002
Stage II	1.700 (1.479-1.953)	<0.001	1.435 (1.131-1.820)	0.003
Stage III	2.644 (2.301-3.038)	<0.001	2.032 (1.603-2.575)	<0.001
Stage IV	4.981 (4.333-5.727)	<0.001	3.480 (2.742-4.417)	<0.001
Gender (female: ref)				
male	1.092 (1.065 - 1.119)	<0.001	1.281 (0.956-1.717)	0.097
Stage Category*gender				
Stage I *male			1.007 (0.741-1.370)	0.963
Stage II*male			0.837 (0.622-1.127)	0.241
Stage III*male			0.848 (0.631-1.139)	0.274
Stage IV*male			0.851 (0.633-1.146)	0.288
Metastases. Recurrence (no metastases and no recurrence as ref)				
Metastases	2.342 (2.269 – 2.418)	<0.001	1.726 (1.666 -1.789)	<0.001
Recurrence	1.329 (1.193 – 1.482)	<0.001	1.269 (1.137-1.416)	<0.001
Metastases and recurrence	1.567 (1.354 – 1.814)	<0.001	1.177 (1.015-1.364)	0.031
No recurrence^1^	1.989 (1.932 – 2.050)	<0.001	2.038 (1.976-2.102)	<0.001
Histological type (ref: Adenocarcinoma)				
Other	1.203 (1.166-1.242)	<0.001	1.165 (1.128-1.202)	<0.001
Squamous cell carcinoma	1.234 (1.154-1.318)	<0.001	1.240 (1.160-1.326)	<0.001
Other carcinomas	1.179 (1.136-1.225)	<0.001	1.181 (1.137-1.227)	<0.001
Sarcomas	0.330 (0.228-0.478)	<0.001	0.624 (0.431-0.904)	0.013
Total comorbidities count per patient	0.803 (0.795 – 0.811)	<0.001	0.800 (0.787 – 0.812)	<0.001
Comorbidity diagnosis time (ref: cancer diagnosed first)				
Comorbidity diagnosed first	1.436 (1.221-1.689)	<0.001	1.544 (1.497-1.592)	<0.001
Simultaneously	1.087 (0.901-1.313)	0.382	1.673 (1.381-2.027)	<0.001
Comorbidities				
Cancer complications	1.754 (1.491-2.062)	<0.001	2.155 (1.817-2.556)	<0.001
Alcohol disorder	1.011 (0.908-1.127)	0.834	1.162 (1.039-1.300)	0.009
Pregnancy related	0.904 (0.806-1.014)	0.086	1.407 (1.246-1.588)	<0.001
Obesity	1.152 (0.945-1.405)	0.162	1.344 (1.101-1.641)	0.004
Malnutrition	0.896 (0.729-1.102)	0.300	1.327 (1.075-1.639)	0.009
Gastrointestinal diseases	0.769 (0.745-0.793)	<0.001	0.992 (0.957-1.029)	0.670
Liver. Pancreas diseases	0.687 (0.649-0.727)	<0.001	1.039 (0.976-1.104)	0.230
Cardiovascular diseases	0.754 (0.731-0.777)	<0.001	0.925 (0.893-0.958)	<0.001
Cholecystitis	0.613 (0.578-0.651)	<0.001	0.853 (0.801-0.909)	<0.001
Infectious diseases	0.537 (0.503-0.574)	<0.001	0.813 (0.758-0.872)	<0.001

^1^patients with identified recurrence status, but lacking definite status of metastases presence.

#### 3.8.1 Demographics

Age significantly predicted mortality, with an 17.1% increase in hazard per 10-year increase (p < 0.001). Male gender wasn’t independently associated with mortality (AHR = 1.281; p = 0.097), though this effect was modified by cancer stage (described further).

#### 3.8.2 Cancer stage and interactions

Stage at diagnosis was a strong predictor of survival. Using *In Situ* as a reference AHR for Stage I was 0.684 (p=0.002) showing the lowered risk. Next stages showed higher AHR equal to 1.435 (0.003), 2.032 (p<0.001) and 3.480 (p<0.001) for II, III and IV stages respectively. Stage×sex interaction terms were non-significant (all p>0.24), indicating no evidence that the effect of stage differed by sex.

#### 3.8.3 Metastasis and recurrence

Patients without recurrence/metastasis served as the reference. All other groups showed increased hazard. Notably, a group labelled “no recurrence” (with missing metastasis data) showed a high AHR of 2.038 (p < 0.001), likely due to data gaps in the metastasis data.

#### 3.8.4 Histology

Adenocarcinoma was used as the reference. Other carcinoma types had slightly elevated AHRs, except for the sarcomas group.

#### 3.8.5 Comorbidities and timing of main diagnosis

Timing of non-cancer comorbidities was also informative when compared with “cancer diagnosed first”. Patients whose comorbidity was diagnosed first (AHR = 1.544, p<0.001) or simultaneously with cancer (AHR = 1.673, p<0.001) had higher mortality. At the category level, several comorbidities were independently associated with worse outcomes: cancer complications (AHR = 2.155, p<0.001), pregnancy-related conditions (AHR = 1.407, p<0.001), obesity (AHR = 1.344, p=0.004), malnutrition (AHR = 1.327, p=0.009), and alcohol use disorder (AHR = 1.162, p=0.009). Other categories were not significant (gastrointestinal diseases AHR = 0.992, p=0.670; liver/pancreas diseases AHR = 1.039, p=0.230). Notably, several comorbidity groups showed lower hazards-cardiovascular diseases (AHR = 0.925, p<0.001), cholecystitis (AHR = 0.853, p<0.001), and infectious diseases (AHR = 0.813, p<0.001). Consistent with this pattern, a higher count of comorbidity categories was associated with a lower hazard (per additional category AHR = 0.800, p<0.001), which likely reflects differential recording/ascertainment rather than a protective biological effect.

## 4 Discussion

This is the first nationwide study in Kazakhstan to assess gastric cancer trends, outcomes, and predictors. Data was taken from national electronic health records from 2012 - 2023. Despite a modest decline in incidence (ASIR 13.6/100,000 in 2023), the 5-year survival remains low at 17.1%, and 82.7% of patients died during the study period. It indicates persistent challenges in detection and treatment.

### 4.1 Burden and trends of gastric cancer

According to publicly available reports in local websites, journals in 2024^th^ year in Kazakhstan there were 2927 new cases of the stomach cancer, and 67% are of male gender. The highest incidence is identified within the 50–74 years old age group (11). These patterns are broadly consistent with our cohort, supporting the plausibility of our age–sex distributions and suggesting near–population-level coverage. Regionally, North Kazakhstan reported incidence rates of 16.1 per 100,000 in the first nine months of 2022 and 16.2 per 100,000 in 2021 (22), whereas our nationwide estimates for the full year were 14.56 and 14.58 per 100,000, respectively. Given that North Kazakhstan is recognized for a higher oncologic burden than many other regions, the elevated regional rates are expected and align with known geographic variation rather than indicating under-ascertainment in our dataset.

In 2023, Kazakhstan’s age-standardized incidence rate (ASIR) was found to be 13.6 per 100,000, higher than in the U.S. (4.2) and Europe (8.1). However, it was lower than in East Asian countries such as China (20.6), Japan (48.1 for males) and Mongolia (47.2 for males) (23, 24). In Kyrgyz Republic in 2016^th^ year the stomach cancer incidence was 10.9 per 100,000 and 10,0 per 100,000 people in 2017^th^ year (25). While in these years in Kazakhstan incidence was 17.4 and 16.8 per 100,000 people cases respectively, showing higher incidence. In other neighbor country, Uzbekistan, the incidence is even lower, despite the fact it is ranked first out of oncological diseases among men, and fifth among women. In 2020–2023 years, incidence was 5.3 cases per 100,000 individuals, and mortality rate ranging between 3.8 to 4.0 death per 100,000 people (26). In Kazakhstan in 2020–2023 years incidence ranged between 14.2 to 13.6 cases per 100,000 people, and mortality from 11.7 to 8.7 deaths per 100,000 individuals. It is seen that compared with neighborhood countries, Kazakhstan has higher incidence and mortality within the same comparison years. Also, despite regional variation, Kazakhstan’s ASIR remains ~1.5 times higher than the global average that is equal to 9.2 per 100,000 (24). Mortality followed a declining trend, with ASMR falling from 16.6 to 8.7, nevertheless survival outcomes remain poor. These findings highlight significant deficits in early detection, access to care, and treatment effectiveness.

### 4.2 Late diagnosis and screening gaps

**Figure 8:**
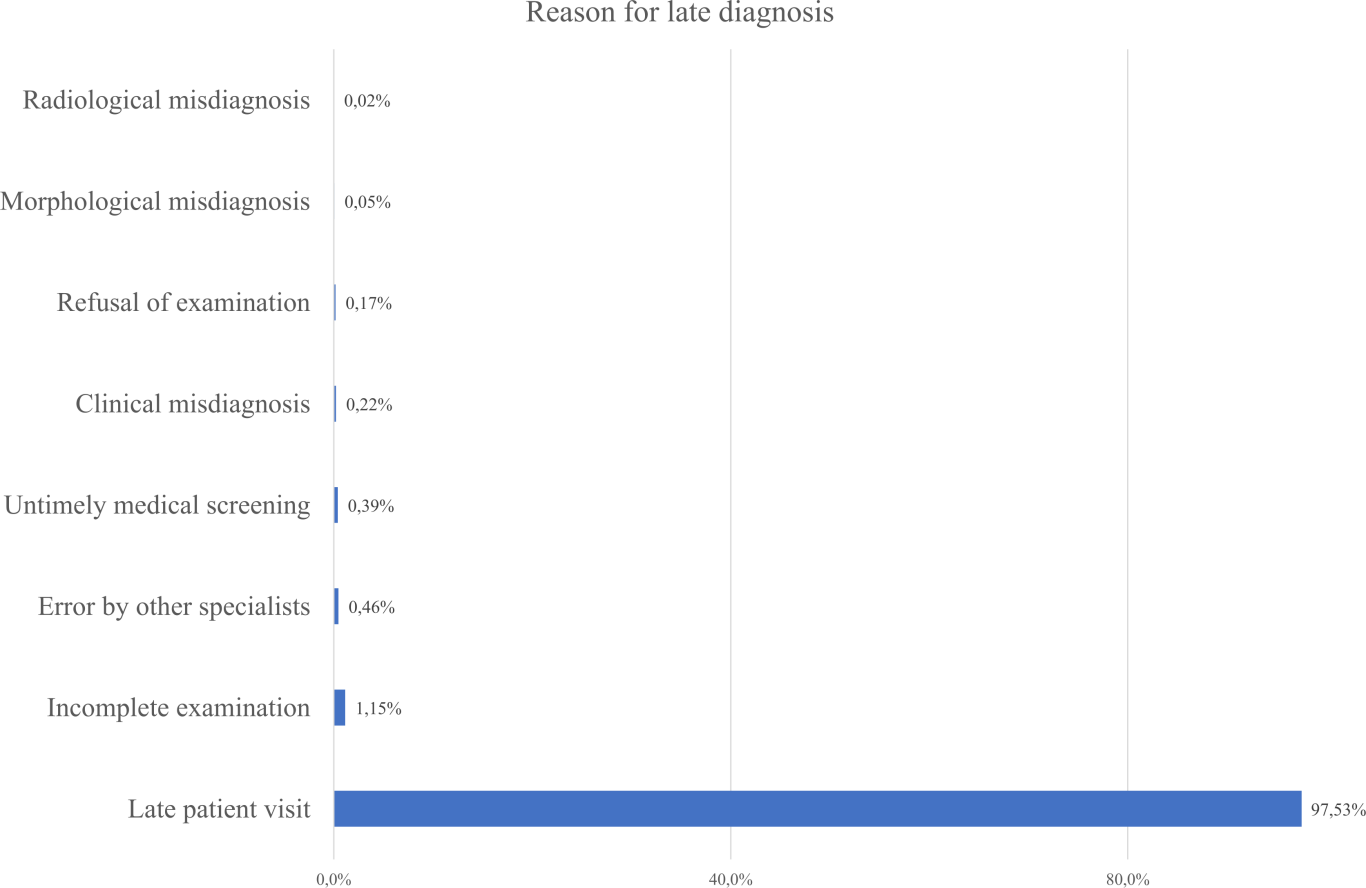
Reported reasons for delayed gastric cancer diagnosis based on the present data in the database.

**Figure 9:**
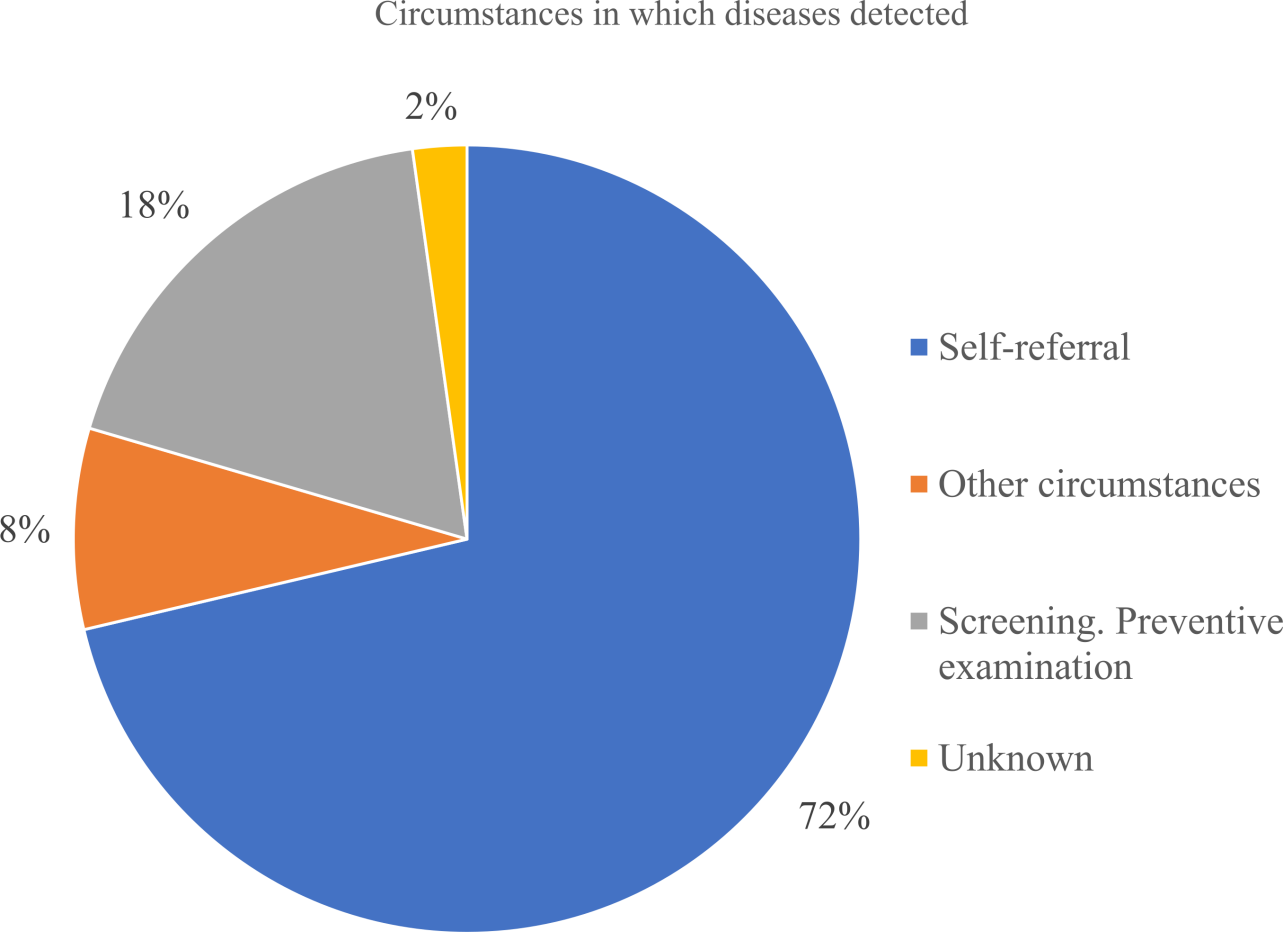
Circumstances in which gastric cancer was detected according to registered reasons in the database.

As seen from **Figure 8** most patients were diagnosed at Stage III (33.5%), followed closely by Stage II and Stage IV diagnosis. Combined - 87% of patients were diagnosed at Stage II or later, reflecting delayed detection and the main reason was delayed healthcare-seeking behavior. Only 18% of cases were identified through screening, while 72% were detected due to patient’s self-referral to medical practitioners. It indicates low participation in organized screening programs (**Figure 9**). Nevertheless, the proportion of Stage IV diagnoses decreased by around 10% over the study period – it can be linked to the 2012 implementation of Kazakhstan’s colorectal cancer screening program.

International comparisons emphasize the value of early detection. Japan and Korea, with long-standing nationwide screening, report 5-year survival rates around 69%, compared to Kazakhstan’s 17.1% (27, 28). Modest screening efforts in China have led to increased survival from 30.2% to 35.9% (29). In Kazakhstan, survival by stage remains much lower than in regional neighbors. For instance, Stage II survival was 22.1% in our cohort, versus 77.6% in Japan and 85.4% in China - showing lack of earlier diagnosis and curative treatment (30, 31).

### 4.3 Predictors of survival and risk stratification

Multivariable analysis identified several key predictors of survival, including age, gender, cancer stage, metastasis, recurrence, histology, and comorbidities. Hazard ratios rose significantly with advancing stage: Stage IV had an AHR >4.0 compared to *In Situ*. Presence of recurrence or metastases was associated with nearly 30% mortality, consistent with global data on poor prognosis in advanced disease (29, 32, 33).

Adenocarcinoma accounts for ~90% of gastric cancers (29), but in our dataset it was recorded in only 61.6% of cases. This discrepancy likely reflects incomplete morphology coding: 1,975 records lack morphology, and the broad “other” category aggregates heterogeneous entities - germ cell tumors (n=5), dendritic cell tumors (n=1), leukemia (n=2), lymphomas (n=50), neuroendocrine carcinomas (n=185), “other tumors” (n=6,092), and central nervous system tumors (n=2). The large “other tumors” subgroup (n=6,092) is of uncertain origin; some proportion may in fact be adenocarcinoma, but this cannot be verified from available fields.

So, the most prevalent histology type adenocarcinoma (61.6%) in our database, was associated with worse outcomes. Rarer types like sarcomas and other tumors group (neuroendocrine, leukemia, lymphoma) had lower sample sizes but showed varying prognoses. Although gastric tumors with non-carcinoma histology were staged using the same system in the national registry, we acknowledge that these subtypes follow distinct biological behaviors. Their inclusion may introduce heterogeneity. However, due to their low frequency, we believe the impact on overall survival estimates was limited.

Occupational status was not included in the multivariable Cox regression model due to its lack of statistical significance in univariate analysis. Also, data completeness and heterogeneity across categories was a concern. However, given its relevance to cancer disparities, descriptive analysis of occupation is presented in **Figure 4**. It highlights socioeconomic patterns in disease distribution. Workers in hazardous industries (e.g., mining, manufacturing) showed the second-highest disease prevalence after pensioners, consistent with findings from other studies (34).

### 4.4 Role of comorbidities

Comorbidities strongly influenced survival. Malnutrition, obesity, alcohol use disorder, pregnancy-related conditions, and cancer complications were the strongest negative predictors. These findings align with studies from China and Iran, where both malnutrition and obesity increased gastric cancer risk and worsened outcomes (35–37). A recent meta-analysis also linked alcohol use to increased gastric cancer risk (OR = 1.20), consistent with our findings (AHR = 1.23) (35).

Unexpectedly, some comorbidities - including cardiovascular (AHR = 0.925), cholecystitis (AHR = 0.853), and infectious diseases (AHR = 0.813) - were associated with reduced hazard of death. These associations are counterintuitive. Also, it was seen that with increased number of comorbidities registered, the less hazard was identified. These findings justify the issue in registry rather than the biological effect. First, residual confounding may be present if patients with well-documented comorbidities had better healthcare access or were more closely monitored. Second, immortal time bias is a plausible explanation: patients had to survive long enough after their cancer diagnosis to be diagnosed with these comorbidities, artificially lowering their observed hazard. Third, misclassification is possible due to limitations in registry coding, particularly regarding the timing and severity of conditions. Moreover, as seen from **Table 1** the “0 or missing” group had the highest percentage of the data with the highest prevalence in later stages (Stage III, IV). The more comorbidities one patient had, the lower was the stage at the registration. This pattern most like is the reason for this so-called protective behavior of the comorbidities.

While we acknowledge these limitations, sensitivity analyses excluding comorbidities diagnosed after the primary cancer event are warranted in future work to clarify these associations.

### 4.5 Treatment data gaps and clinical practice in Kazakhstan

A major limitation of this study is the absence of full data on treatments such as surgery, chemotherapy, and radiotherapy. The UNEHS does not consistently capture treatment modalities in structured formats suitable for large-scale survival analysis. As a result, we were unable to assess the impact of treatment type, timing, or completion on survival outcomes.

While our analysis could not include treatment variables, it is important to contextualize survival outcomes by considering the national gastric cancer treatment guidelines and potential implementation challenges. The national clinical guidelines for gastric cancer in Kazakhstan largely align with international standards, recommending perioperative chemotherapy (e.g., FLOT, XELOX, or fluorouracil-based regimens), radical surgical resection with D2 lymphadenectomy, and adjuvant therapy based on tumor stage and histology. Standard protocols involve intensive chemotherapy regimens, such as 8 cycles of FLOT or XELOX, and sometimes chemoradiation, with cycles repeated every 2 to 3 weeks over several months.

In comparing with results obtained from the database, the stage pattern (more surgery in I–II; very little in IV) matches the protocol direction. However, the overall rate of “no surgery/missing” is much higher than expected for a curative-intent population and justifies the possibility of “missing” data, rather than “no operation” group purely. The same comes to non-surgical treatment. The protocol expects peri-operative chemotherapy for T3/N+ (many stage II–III) and palliative chemotherapy for most stage IV patients. **Table 2** likely understates chemotherapy use because of very high missingness, especially in stage IV where chemo should be common unless patients are unfit. So, overall, we see that even the limited data is consistent with protocol, however due to gaps we consider treatment variables as unreliable for primary modeling.

Importantly, the protocol outlines detailed drug schedules but lacks structured pathways for post-treatment rehabilitation or functional recovery. Most patients transition to lifelong dietary management and basic imaging follow-up, but no formal physical therapy, nutritional counseling, or psychological support is integrated into routine survivorship care.

### 4.6 Post-treatment outcomes and quality of life

**Figure 10:**
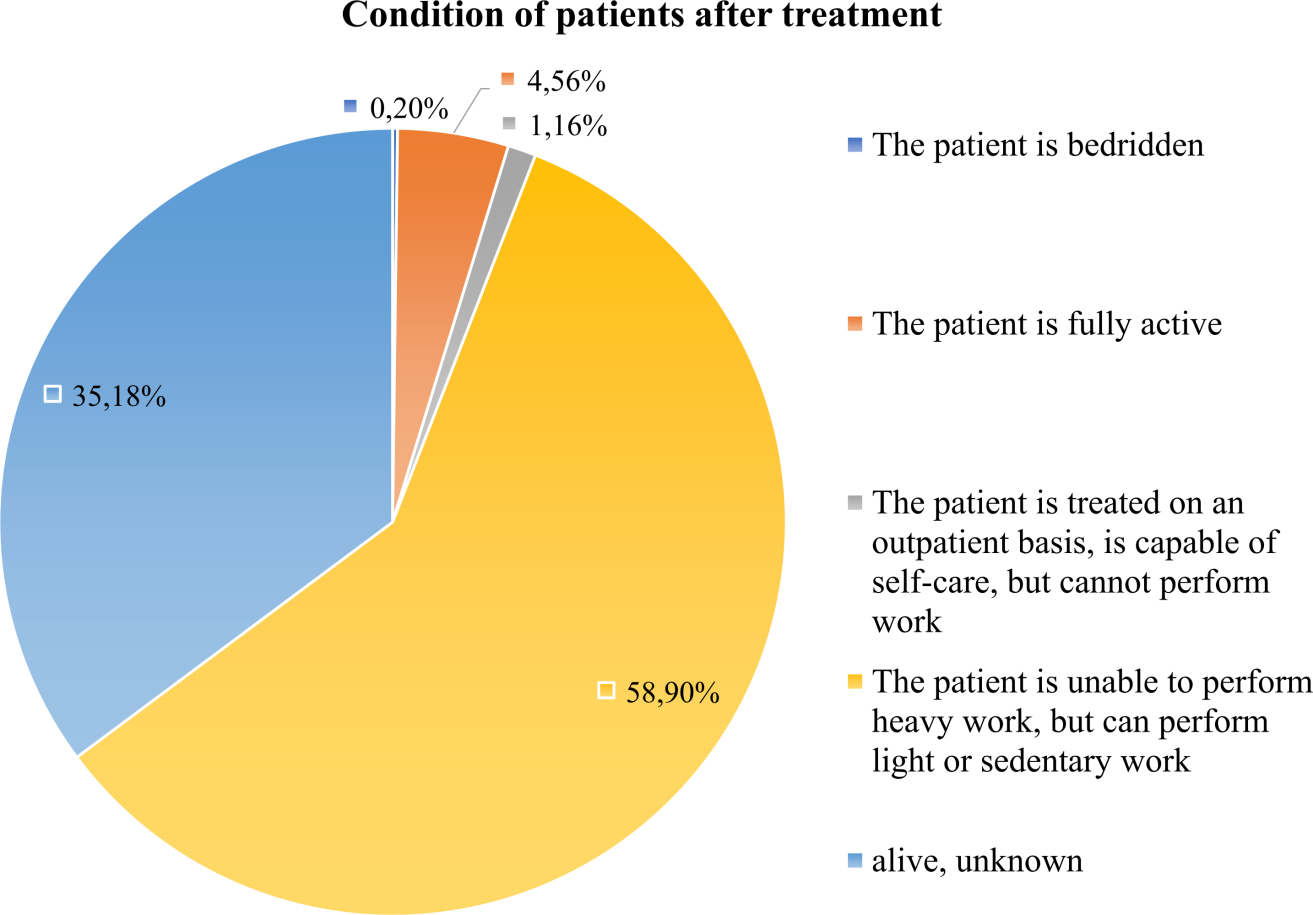
Functional status of gastric cancer post-treatment survivors. This figure shows the distribution of functional/physical status among survived patients after treatment. Most survivors reported limited or sedentary activity, while only a minority regained full physical functioning.

Post-treatment recovery and follow-up remain inadequate. Oncological registry had information on the condition of patients. From 17% of the whole cohort that was alive, only 4.6% of patients returned to full physical activity, while the majority were limited to light or sedentary work. 35.2% were lost to follow-up (**Figure 10**). System lacks structured survivorship care, with minimal nutritional guidance, psychological support, or recurrence monitoring. Given the known association between nutritional status, lifestyle factors, and gastric cancer outcomes, integrating comprehensive follow-up services is critical to improving quality of life and reducing recurrence (38, 39). Integrating supportive care and rehabilitation into national protocols could help improve long-term performance outcomes and quality of life.

### 4.7 Strengths and limitations

The strength of this study is the population-based registry, which provides sufficiently large, representative information with detailed staging, histology, and survival data. Also, it provides new statistically significant data on gastric cancer epidemiology for 2012–2023 years essential for further analysis and public health policy. However, limitations include missing information on treatment modalities, H. pylori infection status, smoking, dietary patterns, socioeconomic status (SES), incomplete histology classification, and lack of cause-specific mortality. These unmeasured factors could confound observed associations (e.g., age/stage effects partly mediated by H. pylori or smoking; survival differences influenced by SES or adherence). Leaving out treatment can bias our survival estimates because treatment is tied to baseline fitness and disease severity (classic confounding by indication), and if we treat post-baseline therapy as a baseline covariate, we risk immortal-time bias. UNEHS captures only clinically significant events - hospitalizations, outpatient visits, and official follow-up registrations. As a result, stomach cancer patients with poor access to primary care may never enter the system and could be missing from our dataset. Staging errors and lack of post treatment updates further reduce accuracy for treatment monitoring and epidemiological surveillance.

## 5 Conclusion

This first nationwide analysis of gastric cancer in Kazakhstan reveals persistently poor survival despite declines in incidence and mortality between 2012 and 2023. Over 87% of cases were diagnosed at Stage II or higher, with survival significantly affected by cancer stage, recurrence, nutritional status, and comorbidities. Cardiovascular and gastrointestinal comorbidities were most common. A key limitation of this study is the lack of data on surgical, chemotherapeutic, or radiotherapeutic treatment. To improve prognosis, future research should incorporate detailed treatment data and refine prognostic models. To reduce this limitation in future work, the registry could (i) when possible or were done add H.Pylori results into the database; (ii) add structured smoking fields, add optional lifestyle modules for alcohol, diet and SES. These enhancements would enable better confounding control and support more causal interpretations. Strengthening registry accuracy, expanding screening - especially for older adults with known risk factors - and ensuring timely diagnosis are essential. Investment in equitable oncology services, implementing screening, and structured post-treatment care is critical to reduce mortality and improve outcomes for gastric cancer patients nationwide.

## Data Availability

The raw data supporting the conclusions of this article will be made available by the authors, without undue reservation.
