# Correction: Survival disparities and predictors in gastric cancer: a population-based study from Kazakhstan (2012–2023)

**DOI:** 10.3389/fonc.2026.1779632

**Published:** 2026-01-15

**Authors:** Aidana Rakhmankulova, Yesbolat Sakko, Altay Kerimkulov, Meiram Mamlin, Sanzhar Shalekenov, Dinara Zharlyganova, Almira Manatova, Zhuldyz Kuanysh, Zhandos Burkitbayev, Abduzhappar Gaipov

**Affiliations:** 1 Department of Biomedical Sciences, School of Medicine, Nazarbayev University, Astana, Kazakhstan; 2 Department of Multidisciplinary Surgery, National Research Oncology Center, Astana, Kazakhstan; 3 Department of Science, National Research Oncology Center, Astana, Kazakhstan; 4 Department of Medicine, School of Medicine, Nazarbayev University, Astana, Kazakhstan

**Keywords:** gastric cancer, survival, epidemiology, risk factors, mortality, cancer stage, comorbidities, Kazakhstan

An incorrect **Funding** statement was provided. The correct funder is Ministry of Science and Higher Education of the Republic of Kazakhstan. The correct **Funding** statement reads:

“This research was funded by the Committee of Science of the Ministry of Science and Higher Education of the Republic of Kazakhstan grant number BR24992950 (“Creation and Implementation of Innovative Treatment Methods for Oncological Diseases”)”.

The original version of this article has been updated.

